# Supplementary material for: Lauric Acid Induces Apoptosis of Rice Sheath Blight Disease Caused by Rhizoctonia solani by Affecting Fungal Fatty Acid Metabolism and Destroying the Dynamic Equilibrium of Reactive Oxygen Species
Source: J Fungi (Basel). 2022 Feb 1;8(2):153. doi: 10.3390/jof8020153 (PMC8875428; doi:10.3390/jof8020153)
Supplement: Supplementary file 1 [file jof-08-00153-s001.zip › Supplementary Figures S1-S3.pdf]

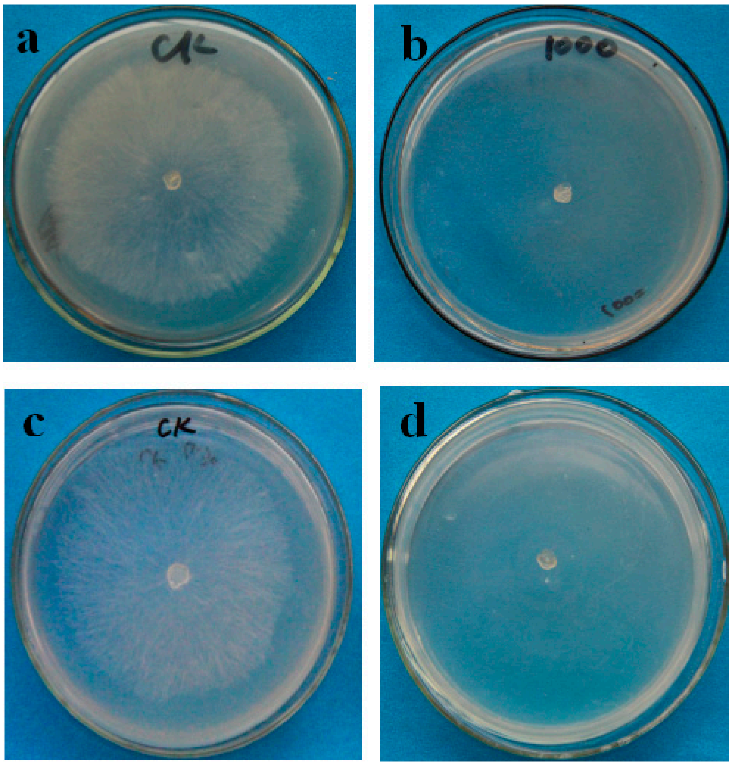

Figure S1. The mode action of lauric acid against *R. solani*

a: 0 µg/mL, **b**: 1000 µg/mL, **c**: the transfer of 0 µg/mL, **d**: the transfer of 1000 µg/mL

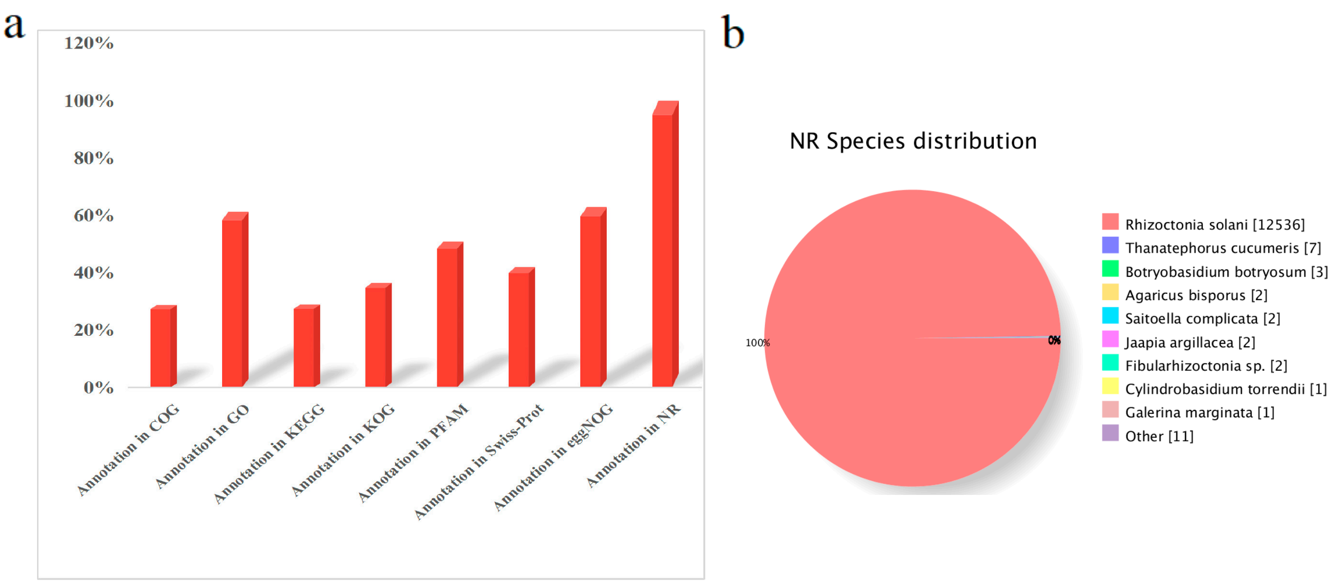

Figure S2. Characteristics of similarity search against Nr databases. a. Similarity distribution of the best BLAST hits for each unigene. **b.** Species distribution of the top BLAST hits for each unigene in Nr database is shown as the percentage of the total homologous sequences with an

E-value of at least $1.0E^{-5}$.

a

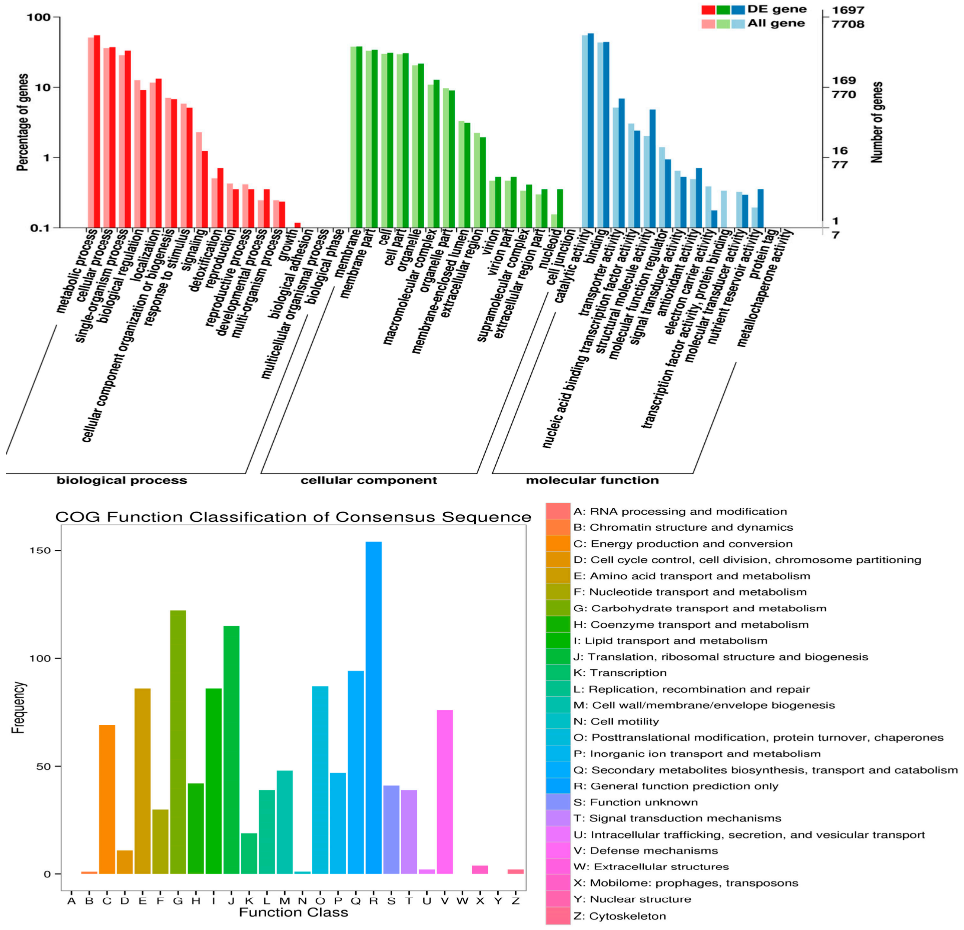
DE gene
All gene
1697
7708
169
770
16
77
1
7
Number of genes
Percentage of genes
100
10
1
0.1
metabolic process
cellular process
single-organism process
biological regulation
localization
cellular component organization or biogenesis
response to stimulus
signaling
detoxification
reproduction
reproductive process
developmental process
multi-organism process
growth
biological adhesion
multicellular organismal process
biological phase
membrane
membrane part
cell
cell part
organelle
macromolecular complex
organelle part
membrane-enclosed lumen
extracellular region
virion
virion part
supramolecular complex
extracellular region part
nucleoid
cell junction
catalytic activity
binding
transporter activity
nucleic acid binding transcription factor activity
structural molecule activity
molecular function regulator
signal transducer activity
antioxidant activity
electron carrier activity
transcription factor activity, protein binding
molecular transducer activity
nutrient reservoir activity
protein tag
metallochaperone activity
biological process
cellular component
molecular function
COG Function Classification of Consensus Sequence
Frequency
150
100
50
0
A B C D E F G H I J K L M N O P Q R S T U V W X Y Z
Function Class
A: RNA processing and modification
B: Chromatin structure and dynamics
C: Energy production and conversion
D: Cell cycle control, cell division, chromosome partitioning
E: Amino acid transport and metabolism
F: Nucleotide transport and metabolism
G: Carbohydrate transport and metabolism
H: Coenzyme transport and metabolism
I: Lipid transport and metabolism
J: Translation, ribosomal structure and biogenesis
K: Transcription
L: Replication, recombination and repair
M: Cell wall/membrane/envelope biogenesis
N: Cell motility
O: Posttranslational modification, protein turnover, chaperones
P: Inorganic ion transport and metabolism
Q: Secondary metabolites biosynthesis, transport and catabolism
R: General function prediction only
S: Function unknown
T: Signal transduction mechanisms
U: Intracellular trafficking, secretion, and vesicular transport
V: Defense mechanisms
W: Extracellular structures
X: Mobilome: prophages, transposons
Y: Nuclear structure
Z: Cytoskeleton

b

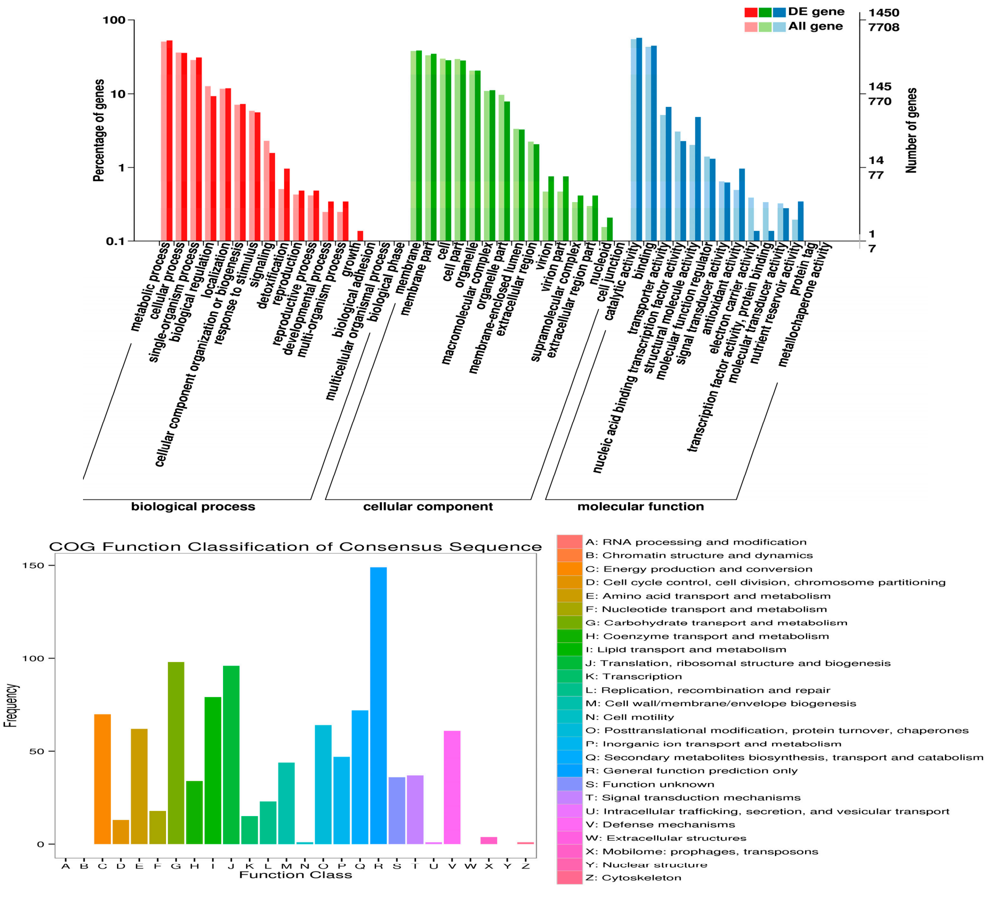
DE gene
All gene
1450
7708
145
770
14
77
1
7
Number of genes
Percentage of genes
100
10
1
0.1
metabolic process
cellular process
single-organism process
biological regulation
localization
cellular component organization or biogenesis
response to stimulus
signaling
detoxification
reproduction
reproductive process
developmental process
multi-organism process
growth
biological adhesion
multicellular organismal process
biological phase
membrane
membrane part
cell
cell part
organelle
macromolecular complex
organelle part
membrane-enclosed lumen
extracellular region
virion
virion part
supramolecular complex
extracellular region part
nucleoid
cell junction
catalytic activity
binding
transporter activity
nucleic acid binding transcription factor activity
structural molecule activity
molecular function regulator
signal transducer activity
antioxidant activity
electron carrier activity
transcription factor activity, protein binding
molecular transducer activity
nutrient reservoir activity
protein tag
metallochaperone activity
biological process
cellular component
molecular function
COG Function Classification of Consensus Sequence
Frequency
150
100
50
0
A B C D E F G H I J K L M N O P Q R S T U V W X Y Z
Function Class
A: RNA processing and modification
B: Chromatin structure and dynamics
C: Energy production and conversion
D: Cell cycle control, cell division, chromosome partitioning
E: Amino acid transport and metabolism
F: Nucleotide transport and metabolism
G: Carbohydrate transport and metabolism
H: Coenzyme transport and metabolism
I: Lipid transport and metabolism
J: Translation, ribosomal structure and biogenesis
K: Transcription
L: Replication, recombination and repair
M: Cell wall/membrane/envelope biogenesis
N: Cell motility
O: Posttranslational modification, protein turnover, chaperones
P: Inorganic ion transport and metabolism
Q: Secondary metabolites biosynthesis, transport and catabolism
R: General function prediction only
S: Function unknown
T: Signal transduction mechanisms
U: Intracellular trafficking, secretion, and vesicular transport
V: Defense mechanisms
W: Extracellular structures
X: Mobilome: prophages, transposons
Y: Nuclear structure
Z: Cytoskeleton

c

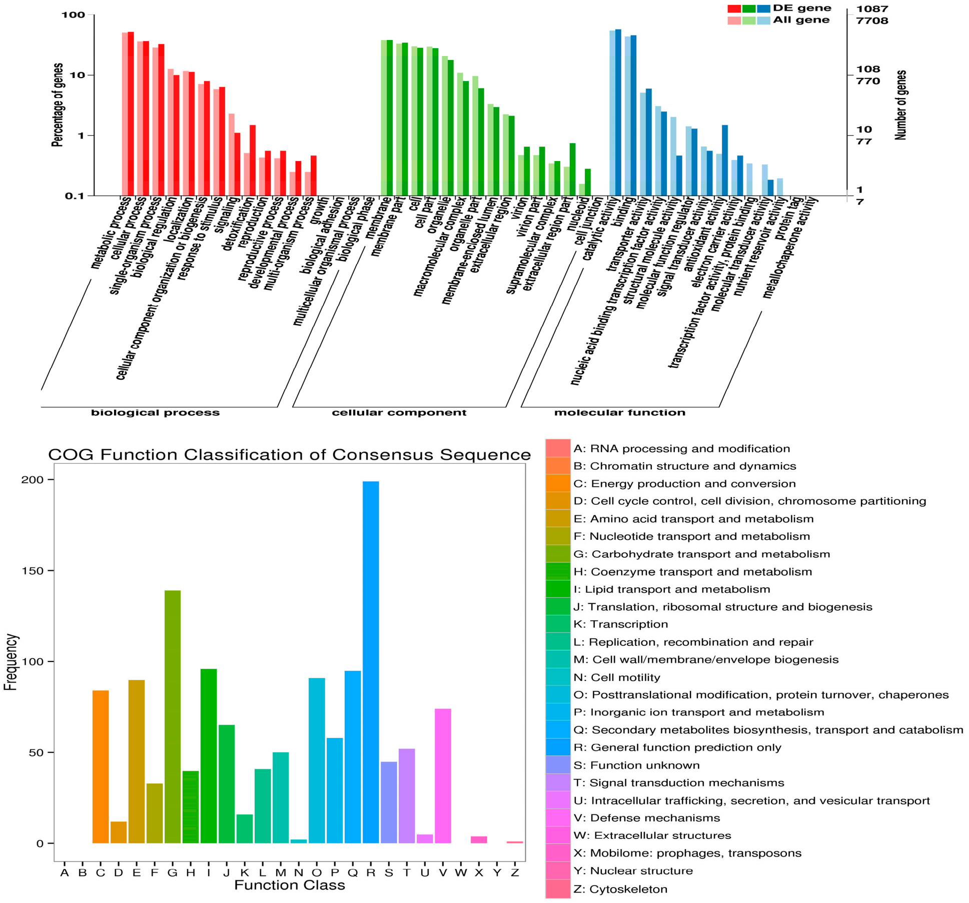

d

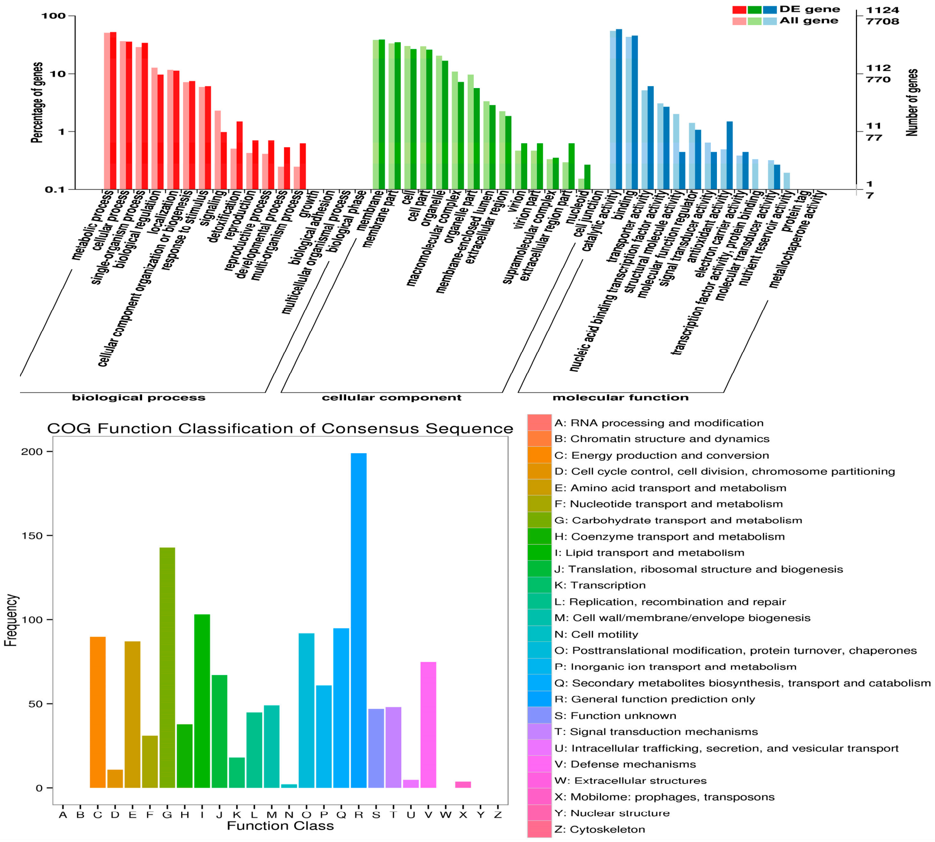

Figure S3. GO/COG enrichment analysis of DEGs between different samples. The most

enriched GO terms are shown. The calculated *P*-value underwent a Bonferroni correction and the threshold of the corrected *P*-value was less than 0.05. **a**: GO/COG enrichment analysis of DEGs in rice sheath blight responding to lauric acid for 3 h; **b**:GO/COG enrichment analysis of DEGs in rice sheath blight responding to lauric acid for 6 h; **c**: GO/COG enrichment analysis of DEGs in rice sheath blight responding to lauric acid for 18 h; **d**: GO/COG enrichment analysis of DEGs in rice sheath blight responding to lauric acid for 24 h.
